# Cognitive rehabilitation following traumatic brain injury

**DOI:** 10.1590/S1980-57642011DN05010004

**Published:** 2011

**Authors:** Fabio Rios Freire, Fernanda Coelho, Juliana Rhein Lacerda, Marcio Fernando da Silva, Vanessa Tome Gonçalves, Sergio Machado, Bruna Velasques, Pedro Ribeiro, Luis Fernando Hindi Basile, Arthur Maynart Pereira Oliveira, Wellingson Silva Paiva, Paulo Afonso Medeiros Kanda, Renato Anghinah

**Affiliations:** 1 Reference Center of Behavorial and Cognitive Disorders of the Faculty of Medicine, University of São Paulo, São Paulo SP, Brazil.; 2 Laboratório de Mapeamento Cerebral e Integração Sensório-Motora (IPUB/UFRJ), Rio de Janeiro RJ, Brazil.; 3 Psychophysiology Laboratory, Universidade Metodista de São Paulo & High-Resolution EEG Section, Division of Neurosurgery, University of São Paulo Medical School, São Paulo SP, Brazil.

**Keywords:** TBI, traumatic brain injury, cognitive rehabilitation

## Abstract

Annually, some 500,000 people are hospitalized with brain lesions acquired after traumatic brain injury (TBI) in Brazil. Between 75,000 and 100,000 individuals die within hours of the event and 70,000 to 90,000 evolve to irreversible loss of some neurological function. The principal causes of TBI include motor vehicle accidents (50%), falls (21%), assaults and robberies (12%) and accidents during leisure activities (10%). Within this context, cognitive rehabilitation, a clinical area encompassing interdisciplinary action aimed at recovery as well as compensation of cognitive functions altered as a result of cerebral injury, is extremely important for these individuals. Therefore, the aim of this study was to review the basic concepts related to TBI, including mechanisms of injury, severity levels of TBI, the most common findings in moderate and severe TBI survivors, and the most frequent cognitive impairments following TBI, and also to discuss the strategies used to handle patients post-TBI. The study results yielded relevant information on a structured cognitive rehabilitation service, representing an alternative for patients and families afflicted by TBI, enabling the generation of multiple research protocols.

## Abstract (pt)

Anualmente, 500 mil pessoas são hospitalizadas com lesão cerebral adquirida após traumatismo crânio-encefálico (TCE) no Brasil. Setenta e cinco a cem mil pessoas morrem poucas horas após o evento e 70 a 90 mil evoluem para perda irreversível de alguma função neurológica. Entre as principais causas de TCE estão os acidentes automobilísticos (50%), quedas (21%), assaltos e roubos (12%) e atividades de lazer (10%). Dentro deste contexto, a reabilitação cognitiva, uma área clínica de atuação interdisciplinar em busca de recuperação, tanto quanto a compensação de alterações das funções cognitivas resultantes de lesão cerebral, é extremamente importante para estes indivíduos. Portanto, neste estudo, foram revisados os conceitos básicos relacionados ao TCE, tais como os mecanismos de lesão, os níveis graves de TCE, os achados mais comuns em sobreviventes de TCE moderado e grave e as deficiências cognitivas mais comuns após TCE e discutidas as estratégias utilizadas para lidar com pacientes pós-TCE. Como resultado, nosso estudo irá fornecer informações relevantes relacionadas com um serviço de reabilitação cognitiva estruturada e, certamente, irá oferecer uma alternativa para pacientes e famílias vítimas de TCE e, portanto, gerar múltiplos protocolos de pesquisa.

Traumatic brain injury (TBI) is a nondegenerative, noncongenital insult to the brain from an external mechanical force, possibly leading to permanent or temporary impairment of cognitive, physical, and psychosocial functions, with an associated diminished or altered state of consciousness.^1^ The definition of TBI has not been consistent and tends to vary according to specialties and circumstances. The term brain injury is often used synonymously with head injury, which may not be associated with neurologic deficits. The definition has also been problematic due to variations in inclusion criteria.^2^

Both American and Brazilian data indicate that about 500,000 people suffer TBI annually, with 20% afflicted with moderate or severe TBI. According to this data, 80% of people who suffered mild TBI can return to work, whilst only 20% of moderate, and 10% of victims of severe, TBI patients can return to their daily routine.^3,4^

In this context, cognitive rehabilitation, a clinical area encompassing interdisciplinary action aimed at recovery as well as compensation of cognitive functions altered as a result of cerebral injury, is extremely important for these individuals.^5^ The aim of a cognitive rehabilitation program is to recover an individual’s ability to process, interpret and respond appropriately to environmental inputs, as well as to create strategies and procedures to compensate for lost functions that are necessary in familial, social, educational and occupational relationships.^2^ In general, the cognitive rehabilitation programs tend to focus on specific cognitive domains, such as memory, motricity, language and executive functions. By contrast, the focus of compensatory training procedures is generally on making environmental adaptations and changes to provide greater autonomy for patients. Successful cognitive rehabilitation programs are those whose aim is both recovery and compensation based on an integrated and interdisciplinary approach.^6^

The purpose of this study was to review the basic concepts related to TBI, including mechanisms of injury, severity levels of TBI, the most common findings in moderate and severe TBI survivors, and the most frequent cognitive impairments following TBI, and also to discuss the strategies used to handle patients post-TBI. Within this context, the importance of an interdisciplinary rehabilitation for TBI is underlined. According to the above topics, a strategic search of the studies held on the main data bases was carried out. The computer-based search covered the following databases: Pubmed/Medline, ISI Web of Knowledge, Cochrane data base and Scielo. The search terms, physical therapy, occupational therapy, psychology, phonoaudiology, recovery, rehabilitation, traumatic brain injury, brain injury and TBI in combination with cognitive rehabilitation, were employed. Papers and book chapters published in Portuguese, Spanish and English, conducted from 2001 to 2010 were preferentially reviewed.

## Mechanism of injury

The mechanisms and consequences that lead to brain injury vary depending on the type of trauma. Injury occurs when the skull is struck by an object or collides against a hard surface resulting in kinetic energy transfer from the object to the head that can cause fractures or lesions in the brain tissue. Motor vehicle accidents and falls from high places often cause such fractures or lesions. Another mechanism constitutes the sudden arrest of head movement, leading to acceleration-deceleration, while the brain remains in its original inertial motion and is suddenly driven off in the opposite direction. In this mechanism the areas of lesion or friction may damage brain tissue that collides against the bone structure. Cortical contusions may also occur as a consequence of tissue abrasion and may result in focal bleeding and edema. An event commonly occurring in car accidents is head trauma caused by collision against the windshield, followed by a rapid backward movement of the head which can cause injury in posterior areas. Such lesions are called coup and contrecoup. Lesions can also occur in the lower portion of the brain surface by traumatic contact against the irregular surface of the skull base, during the acceleration and deceleration movement inside the skull. Orbital and lateral basal portions of the front and temporal lobes are especially vulnerable to this type of lesion.^7^

Another cause of focal lesion occurs with the rupture of small meningeal vessels (due to direct impact leading to skull fracture and extradural hematomas) or brain surface, through the acceleration-deceleration mechanism leading to subdural hematomas. The mechanism and the intensity of the impact of head trauma and the cerebral edema generated by it can cause intraparenchymal hemorrhage and/or edema and lead to infarct areas caused by reduction or discontinuation of cerebral perfusion.^7^

The acceleration-deceleration mechanism can cause neural lesions, and diffuse axonal injury (DAI). The long fiber tracts are more vulnerable to this type of injury. Depending on the location and extent of DAI, extensive brain areas may be affected and could trigger a cascade of damaging processes including failures in axonal transport and diffuse injury, with functional discontinuity of the axon. This process typically occurs in the initial 24 hours following trauma, but may extend further, depending on its severity. The areas most commonly affected by DAI are the medial frontal lobes, the corpus callosum and the superior cerebellar peduncles.^7^

In addition to trauma, other mechanisms may be linked to the sequelae that affect patients, such as surgical manipulation, disruption of the blood-brain barrier with late hemorrhage, and in cases of multiple trauma, shock, cardiac and/or respiratory arrest and anoxia, seizures and post-traumatic hydrocephalus, during clinical evolution.^7^

## Severity levels of TBI

The conscience level and/or coma in the first 24 hours, along with the duration of Post Traumatic Amnesia (PTA), after TBI are the most used references to distinguish TBIs as mild, moderate or severe injuries. The most widely used method to classify TBIs is a scoring system called the Glasgow Coma Scale (GCS), which assesses the ability of eye opening, motor and verbal responses as determiners for evaluation. GCS scores range from 3 to 15, where a score less than or equal to 8 indicates severe injury, from 9 to 12 moderate injury, and 13 to 15 mild injury.^8^

Post Traumatic Amnesia denotes the period spanning from coma and up until the moment the patient recovers memory consistently. This period can be associated with a transient state of disorientation, agitation and behavioral disturbances such as insomnia, agitation, confabulation and, occasionally, serious affective and psychotic symptoms.^9^

One of the most-used scales to evaluate Post Traumatic Amnesia is the GALVESTON Orientation and Amnesia Test. Typically, in post-TBI patients, personal orientation recovers before spatial orientation or the perception of what took place. Temporal orientation is the last to recover.

TBI severity is first rated using the Glasgow scale, coma and Post Traumatic Amnesia period. A TBI is considered severe when the GCS is from 3 to 8, the period of coma is longer than 6 hours and the PTA period is longer than 24 hours. Moderate TBI has a GCS from 9 to 12, coma period of less than 6 hours, and PTA of between 1 and 24 hours. Finally, mild TBI has a GCS of 13 to 15, coma lasting 20 minutes or less, and PTA less than or equal to 1 hour.

Despite the support of the scales, the residual consequences post-TBI tend to occur differently in each patient. However, the majority of those who suffer mild traumas have a recovery process without major complications, and are able to return to their pre-trauma activities. By contrast, the majority of patients who suffer moderate and severe TBI present sequelae and limitations. Nevertheless, a number of mild trauma patients have post-trauma repercussions that require special care and attention from specialized professionals.

**Most common findings in moderate and severe TBI survivors** – This group of patients is characterized by diffuse axonal injury and secondary complication at the site or sites of injury. These patients present loss of consciousness and/or alertness. At this stage, the first approaches are medical, surgical, and/or pharmacological. Other supportive care, physical and respiratory therapy are also considered at this point of intervention.^7^ In addition, speech therapy activities for sensory stimulation are recommended to promote neural plasticity and minimize environmental deprivation.^10^

About 2-15% of patients remain in this state, also known as a vegetative state, for 1 year or more.^11^ Most moderate to severe TBI patients gradually regain stimuli responsiveness (movement, speech) despite persistent erratic and disorganized behavior. The first signs of response are often to vocal and auditory stimuli automatically, which may gradually come under some volitional control. Even without verbal manifestation, the next step may involve motor response to verbal commands. Patients are often agitated during this period. These individuals’ evolution is accompanied by confusion, disorientation, significant attention deficit, disinhibition and impairment of memory (includes PTA). The patient’s comprehension, memory and learning remain impaired regardless of regaining speech.

The next stage experienced by these individuals, usually during post-hospital care or while undergoing outpatient monitoring, is characterized by the recovery of orientation and old memories together with difficulty in acquiring new learning and retaining new memory beyond the PTA. The duration and intensity of these cognitive impairments are directly associated with the intensity of the TBI. The intervention in terms of rehabilitation and cognitive stimulation is at a stage where more intensive efforts are made. Acquisition of basic sensory-motor integration (posture control, head and trunk control, gait acquisition and swallowing), training and recovery or stabilization of Basic Activities of Daily Living (ADL) such as dressing and undressing, personal hygiene and eating, among others, are emphasized in an initial approach. The reestablishment of basic cognitive skills (orientation, attention, memory, receptive and expressive communication) and other motor rehabilitation and ambulatory actions are also pursued. The cognitive stimulation from the interdisciplinary team (various professionals attending the patient in a holistic manner to enhance operations) at this stage is fundamental because the patient is only able to embark on motor training if a minimum attentional state can be maintained which allows the patient to execute and repeat the tasks proposed.^7^

In cases of motor damage, motor rehabilitation and physical therapy clearly play a vital role in the patient’s stabilization and/or recovery. From a cognitive perspective, at this stage temporo-spatial orientation and attention call for redoubled efforts to maximize the communication with the patient so as to improve strategies that use motor and cognitive training. In terms of speech, intervention should promote the structuring of activity, with a highly predictable distraction-free environment and also activities that induce self-monitoring.

The biggest hurdle at this stage stems from the fact that most patients have significant attentional deficit that limits the role of comprehensive rehabilitation.

After the most critical phase of hospital management, most patients return home. Although some patients manage to regain some degree of independence in their self-care, they are still incapable of applying critical thinking to decision-making processes, providing for the needs of their families, or continuing work, school or social activities, which can cause difficulties in family relationships and result in a poor quality of life for patients and their relatives. Moreover, patients may manifest mood alteration and depression.

The rehabilitation of these patients after hospital discharge is aimed at a community integration program with day center resources that provide continuity of patient care with vocational and professional training integrated into the rehabilitation process.^10^

The Rancho Los Amigos levels of cognitive functioning scale is a widely used scale that systematically standardizes the functional level of post-TBI patients in order to establish the possibility of their rehabilitation management.^12^ Another scale of diffuse axonal injury rehabilitation stages developed by Tuel^13^ and modified by Katz^14^ is also useful at this stage.

## Rancho Los Amigos scale (Los Amigos Research and Educational Institute)^12^

- **No response**
  A person at this level will:
  - Not respond to sounds, sights, touch or movement.
- **Generalized response**
  A person at this level will:
  - Begin to respond to sounds, sights, touch or movement;
  - Respond slowly, inconsistently, or after a delay;
  - Respond in the same way to what they hear, see or feel.
  Responses may include chewing, sweating, breathing faster, moaning, moving and/or increasing blood pressure.
- **Localized response**
  A person at this level will:
  - Be awake on and off during the day;
  - Make more movements than before;
  - React more specifically to what they see, hear or feel. For example, patient may turn towards a sound, withdraw from pain, and attempt to watch a person move around the room;
  - React slowly and inconsistently;
  - Begin to recognize family and friends;
  - Follow some simple directions such as “Look at me” or “squeeze my hand”;
  - Begin to respond inconsistently to simple questions with “yes” or “no” head nods.
- **Confused-agitated**
  A person at this level will:
  - Be very confused and frightened;
  - Not understand what they feel, or what is happening around them;
  - Overreact to what they see, hear or feel by hitting, screaming, using abusive language, or thrashing about. This is because of the confusion;
  - Be restrained so they do not hurt themselves;
  - Be highly focused on their basic needs; i.e., eating, relieving pain, going back to bed, going to the bathroom, or going home;
  - May not understand that people are trying to help them;
  - Not pay attention or be able to concentrate for only a few seconds;
  - Have difficulty following directions;
  - Recognize family/friends some of the time;
  - With help, be able to do simple routine activities such as feeding themselves, dressing or talking.
- **Confused-inappropriate, non-agitated**
  A person at this level will:
  - Be able to pay attention for only a few minutes;
  - Be confused and have difficulty making sense of things around them;
  - Not know the date, where they are or why they are in hospital;
  - Not be able to start or complete everyday activities, such as brushing their teeth, even when physically able. They may need step-by-step instructions;
  - Become overloaded and restless when tired or when there are too many people around; have a very poor memory, remembering past events from before the accident better than their daily routine or information they have been given since the injury;
  - Try to fill in gaps in memory by making things up; (confabulation)
  - May get stuck on an idea or activity (perseveration) and need help switching to the next part of the activity;
  - Focus on basic needs such as eating, relieving pain, going back to bed, going to the bathroom, or going home.
- **Confused-appropriate**
  A person at this level will:
  - Be somewhat confused because of memory and thinking problems, remembering the main points from a conversation, but forgetting and being confused about the details. For example, they may remember having visitors in the morning, but forget what they talked about;
  - Follow a schedule with some assistance, but becomes confused by changes in the routine;
  - Know the month and year, except when they have a serious memory problem;
  - Pay attention for about 30 minutes, but has trouble concentrating when it is noisy or when the activity involves many steps. For example, at an intersection, they may be unable to step off the curb, watch for cars, watch the traffic light, walk, and talk all at the same time;brush their teeth, get dressed, feed themselves etc., with help;
  - Know when they need to use the bathroom;
  - Do or say things too fast, without thinking first;
  - Know that they are hospitalized because of an injury, but will not understand all the problems they are having;
  - Be more aware of physical problems than thinking problems;
  - Associate their problems with being in the hospital and think they will be fine as soon as they go home.
- **Automatic-appropriate**
  A person at this level will:
  - Follow a set schedule
  - Be able to carry out routine self care without help, if physically able. For example, they can dress or feed themselves independently; have problems in new situations and may become frustrated or act without thinking first;
  - Have problems planning, starting, and following through with activities;
  - Have trouble paying attention in distracting or stressful situations. For example, family gatherings, work, school, church, or sports events;
  - Not realize how their thinking and memory problems may affect future plans and goals. Therefore, they may expect to return to their previous lifestyle or work;
  - Continue to need supervision because of decreased safety awareness and judgement. They still do not fully understand the impact of their physical or thinking problems;
  - Think slower in stressful situations;
  - Be inflexible or rigid, and may be stubborn. However, their behaviors are related to their brain injury;
  - Be able to talk about doing something, but will have problems actually doing it.
- **Purposeful-appropriate**
  A person at this level will:
  - Realize that they have a problem with their thinking and memory;
  - Begin to compensate for their problems;
  - Be more flexible and less rigid in their thinking. For example, they may be able to come up with several solutions to a problem;
  - Be ready for driving or job training evaluation;
  - Be able to learn new things at a slower rate;
  - Still become overloaded with difficult, stressful or emergency situations;
  - Show poor judgement in new situations and may require assistance;
  - Need some guidance making decisions;
  - Have thinking problems that may not be noticeable to people who did not know the person before the injury.

**Cognitive rehabilitation in diffuse axonal injury**
  - Coma: Unresponsive, eyes closed.
  - Vegetative state: No cognitive response, sleep-wake state.
  - Minimally conscious state: Patients wake up if stimulated, respond to some commands, usually in silence.
  - Confusional state: Recovery of speech, amnestic (post traumatic amnesia), severe attentional deficit. Agitated, emotional lability, little interaction with the environment.
  - Post-confusional state: Involves independence, resolution of PTA, cognitive improvement, acquisition of independence in daily self-care. Social relationship improvement and progressive independence development at home.
  - Social competence, community reentry: Regaining of cognitive abilities, personality and independence. Goal Setting. The patients settle back into old routines.

**Most common cognitive impairments following TBI** – The type and degree of the cognitive impairment following TBI can vary widely, depending on the severity and site of injury. If a focal brain injury occurs, the consequence may be similar to an injury caused by a CVA, such as aphasia, apraxia, unilateral neglect or visuospatial dysfunction. However, these are the typical findings following TBI. Due to the mechanisms of acceleration- deceleration that often damage the ventral and lateral regions of the frontal and temporal lobes, the most commonly found sequelae are attention and memory deficit, difficulty in learning new information, resolving problems, planning, as well as problems associated with impulsivity and self-control. Some “subclinical” findings such as change in naming, verbal fluency and auditory discrimination are also reported.

Initially, attention deficits are the most common and severe in the residual stage, usually involving difficulty in maintaining divided attention.

The long-term memory is generally restored, but some individuals continue having difficulties in learning new things and in retaining new information. Working memory is frequently affected including the stages of encoding, storage and retrieval of information. Such changes cause a significant impact on social and vocational reintegration.^15,16^

Many individuals present with amnesic syndrome, more common in those who have gone through periods of hypoxia and anoxia.

Executive functions may also be affected, and are related to frontal lobe damage. In cases of severe frontal injury the patient may be inert, or present no initiative (medial or lateral frontal injury), or exhibit inappropriate behavior and impulsiveness. Many individuals with frontal lobe injury in post-TBI retain many of their skills but are unable to initiate, sequence, organize or monitor their actions so as to meet the targets or goals set.

The most commonly observed language disorders occur in the discursive (tendency to produce irrelevant information and omission of key information), pragmatic (loss in production of inferences, difficulty in formulating arguments) and conversation levels (loss of initiative and maintenance of topics with changes in an inconsistent manner and without signaling). These changes correlate with cognitive impairments in attention, memory and slow mental processing.^10^

The inability to curb impulsive reactions leads to social and family relationship problems. Patients often have poor self-criticism regarding their condition and behavioral changes.

## Strategies to manage post-TBI Patients

**Cognitive function evaluation** – Patients will initially be subjected to a reading test, which consists of reading simple children’s books to evaluate how long they focus on the book. According to the performance and education of each individual, slightly more complex texts will be presented.

After this first test, each patient will be classified according to the Rancho Los Amigos scale and only those who score greater than or equal to 5 (on the scale of 8) will be referred for cognitive rehabilitation.^15^

This is necessary because to be rehabilitated the individual must maintain a minimum time set for the task of greater than 10 minutes.

The next intervention will be to conduct a neuropsychological evaluation, speech and occupational therapy including the Mini Mental State Examination^17,18^ and clock drawing. The neuropsychological assessment includes the evaluation of affective/emotional state,^19-21^ functional activity questionnaire,^22^ batteries of tests of executive functions (Wisconsin sorting card test,^23^ Stroop interference test,^24,25^) as well as other tests including the Rey auditory-verbal learning test,^26^ WAIS III attenttion, digit-symbol and visuo-construtive tests,^27^ trail making test parts A and B,^28^ verbal fluency tests,^29^ and Rey complex figure.^30^ All tests have been previously validated in Brazil with scores for different levels of schooling.

The speech evaluation includes pragmatic assessment, according to precepts of conversational analysis, test of verbal working memory for auditory input (N-Back) language evaluation (Arizona Battery for Communication Disorders - ABCD).^31^

The tests used have been previously validated in Brazil with scores for different levels of schooling except for the “Test of Practical Judgment” (TOP-J), which has no version in Portuguese and the Arizona Battery for Communication Disorders - ABCD, which is undergoing a validation process, and is to be included in our battery. Computerized tests that evaluate response time to a particular task will also be conducted.

Occupational therapy evaluates cognition in order to determine the individual’s capacity to live alone safely and comfortably, and to work or undertake any activity they deem important or meaningful.^32^ Also, the therapy limits the impact of deficits in memory, attention and executive functions in performing activities of daily living. The evaluation of performance in BADL and IADL requires the observation of the individual’s behavior in the context in which they conduct these activities. The information obtained is later used to develop strategies together with the individual and their family which will help minimize the impact of each cognitive loss.

Through the evaluation of cognitive abilities on tasks it is possible to determine the patient’s strengths, limitations and challenges in learning abilities and environmental strategies that will support their daily life.^33,34^ Assessment in Occupational Therapy includes LOTCA.^35,36^

After the neuropsychological, speech and occupational therapy evaluation, individual rehabilitation strategies are developed in conjunction with the interdisciplinary team.

## Tools used in cognitive rehabilitation

Part of the training/rehabilitation will be performed at CEREDIC outpatient units (Reference Center for Cognitive Disturbances - HC/FMUSP) and part by caregivers trained by our team, at the patient’s home. The participant caregivers are given a handout with instructions and the training proposed.

The proposed exercises will be based on the neuropsychological test results, focusing on the most affected attentional subtypes. The behavioral approach entails guidance on caregiver assistance in ADL.

**Attention** – The treatment is usually based on patient engagement in performing repetitive exercises including selective, sustained, alternating and divided attention.

### Sustained attention training

1. Listen to a word sequence and identify when you see a word stimulus, which was previously presented.
2. Understanding of spoken text of a paragraph (originally short and simple), that has progressively increasing difficulty throughout the course of training.
3. Sequencing of numbers in ascending order and / or decreasing verbally.
4. Math Activities - mentally.

### Alternating attention training

1. Exercise in which the patient must identify a previously defined word and word sequence , identifying when it appears in a text or string of words they will listen to, replacing the 1^st^ word, when identified, with the previously given sequence.
2. Tasks with pen and paper, where the patient has to write a number and a letter that complete a sequence written with gaps to be filled.
3. Activities that start with a number, which must be sequentially added or subtracted by the other items that are being presented.

### Selective attention training

1. Any test which has been reported for sustained attention, with a distractor sound or motion associated.
2. Tasks with visual distractors - such as tasks involving drawing with paper and pen (pencil) on a sheet full of tracings and background designs.

### Divided attention training

1. The patient reads a few paragraphs paying attention to their content while the patient looks up the proposed word in advance.
2. The patient completes a test battery of sustained attention training, in which the patient has to respond to verbal or parallel visual stimuli (which can be accomplished by including computerized tests).

## Memory

**Attention** – Discussed in the previous topic.

**Deciphering the meaning of stimuli** – In order to rehabilitate memory, the first aspect is to verify whether the patient maintains sufficient attention on the stimulus or task given (as seen in the previous topic). The second aspect is to assess whether the patient is able to “decipher” the stimulus given, if the patient knows the word or object (verbal or visual stimulus) or can categorize them (words or objects) into any semantic grouping.

The drill used for this type of injury consists of repetition of words. Concurrently, it involves categorization of words such as by asking if a cat is an animal seen at the zoo or by eliciting the rhyme of a given word.

Naturally, a team of speech therapists will evaluate losses involving comprehension and language.

**Memory storage** – This involves learning new tasks or old skills that were lost. When there are bilateral hippocampal lesions, the retention mechanism of long-term learning memory is lost. Processes of verbal repetition and writing are important for this training.

**Evocation** - of old memories – This entails training with pictures or words that are subsequently presented and evoked several times. Repetition, writing, drawing and verbal processes are important in this training. Individuals sustaining frontal lobe injury can remember facts, but not associate them with a context or time of occurrence. Generally, they confabulate based on a pre-existing fact.

In order to rekindle the old memory, a strategy is to repeat the known facts which the patient is unable to remember, using pictures, or by repeating stories until they are remembered. This differs from the bi-hippocampal lesion which preserves old memories (except the period of post-traumatic amnesia) but renders the individual incapable of retaining new learning.

## Training strategies outside the outpatient unit

Initially, the patient undergoes neurological, neuropsychological, speech and occupational therapy evaluation. Once the evaluation is finished, the interdisciplinary team defines the treatment strategy.

Roadmaps will then be developed in each case. The sequence below lists possible training strategies to be developed concurrently with the behavioral training at home.

1. Remember events during the current day (at the end of the day) or for the previous day (in the morning).
2. After recalling the events, write them in a notebook whenever possible.
3. Receive new information - summaries of news about some event or family, read a short informational text.
4. Plan the morning’s, day’s or week’s (Whenever possible) activity.
5. Talk about past events that have been forgotten or are not well contextualized after the accident.
6. Follow the primer of daily activities with attention, executive functions, language and memory activities as well as activities of daily living.
7. Medication approach when appropriate. AChe inhibitors, antidepressants, Ritalin, among others.

The Behavioral Training method is based on the ABA - Applied Behavior Analysis method, based on behaviorism which involves observation, analysis and explanation of the association among environment, human behavior and learning.^37^ Once the behavior is analyzed, an action plan can be implemented to change this behavior. The action plan is based on Operant Conditioning, a term coined by Skinner, which defines a behavior followed by a reinforcing stimulus which results in an increased likelihood of that behavior occurring in the future.^38^

In conclusion, our study yielded relevant information related to a structured cognitive rehabilitation service and represents an alternative for patients and families afflicted by TBI, enabling the generation of multiple research protocols.
